# Trends in and socio-demographic factors associated with caesarean section at a Tanzanian referral hospital, 2000 to 2013

**DOI:** 10.1186/s12939-014-0087-1

**Published:** 2014-10-16

**Authors:** Cecilie Nilsen, Truls Østbye, Anne Kjersti Daltveit, Blandina Theophil Mmbaga, Ingvild Fossgard Sandøy

**Affiliations:** Faculty of medicine and dentistry, Centre for International Health, University of Bergen, Postbox 7804, N-5020 Bergen, Norway; Community and family medicine, Nursing and global health school of medicine, Duke Global Health Institute, Durham, USA; Faculty of medicine and dentistry, Department of Global Public Health and Primary Care, University of Bergen, Bergen, Norway; Kilimanjaro Christian Medical Centre and Kilimanjaro Christian Medical College, Moshi, Tanzania; Faculty of medicine and dentistry, Department of Global Public Health and Primary Care, University of Bergen, Bergen, Norway

## Abstract

**Background:**

Caesarean section (CS) can prevent maternal or fetal complications. Sub-Saharan Africa has the lowest CS levels in the world but large variations are seen between and within countries. The tertiary hospital, Kilimanjaro Christian Medical Centre (KCMC) in Tanzania has had a high level of CS over years. The aim of this study was to examine trends in the socio-demographic background of babies born at KCMC from year 2000 to 2013, and trends in the CS percentage, and to identify socio-demographic factors associated with CS at KCMC during this period.

**Methods:**

This is a registry-based study. The analyses were limited to singletons born by women from Moshi urban and rural districts. The Chi square test for linear trend was used to examine trends in the CS percentage and trends in the socio-demographic background of the baby. The association between different socio-demographic factors and CS was assessed using logistic regression. The analyses were stratified by the mother’s residence.

**Results:**

The educational level of mothers and fathers and the age of the mothers of singletons born at KCMC increased significantly from year 2000 to 2013 both among urban and rural residents. Among 29,752 singletons, the overall CS percentage was 28.9%, and there was no clear trend in the overall CS percentage between 2000 and 2013. In the multivariable model, factors associated with higher odds of CS were: having been referred for delivery, maternal age above 25 and no- or primary education level of the baby’s father. Among rural mothers, no- or primary education, being from the Pare tribe and para 2–3 were also associated with higher odds of CS. Being from the Chagga tribe and high parity were associated with lower odds of CS compared to other tribes and parity 1.

**Conclusions:**

The CS percentage remained high but stable over time. Large variations in CS levels between different socio-demographic groups were observed. The educational level of the parents of babies born at KCMC increased over time, possibly reflecting persistent inequitable access to the services offered at the hospital.

## Background

Providing high quality obstetric care can save the lives of mothers and newborns when complications arise during pregnancy and childbirth [1,2], and caesarean section (CS) is one such essential service. Population-based estimates of CS percentage have been used as an indicator of access to emergency obstetric care [3]. However, CS levels include life-saving interventions as well as cases with no clinical indication [4], and like other major surgery, CS carries the risk of complications and even death for the woman [5]. The World Health Organization (WHO) suggests that the level of CS should range between 5% and 15% [6] although both the upper and the lower limits have been discussed critically by several researchers [7-12]. The average global level of CS is estimated to be about 15% [13], and the lowest levels are found in sub-Saharan Africa (SSA) [10,12,13]. The median CS percentage in facilities which performed CS in SSA in 2004/05 was 13.4% [14]. However facility-based estimates are quite different from population-based estimates as many women do not deliver in facilities in this region [15].

There are large variations in access to maternal health care in SSA, both between and within countries, and between different population subgroups. High levels of CS are no guarantee for equity in access to obstetric care [16]. CS tends to be more common among women in urban than rural areas, and financially better off and higher educated women have considerable higher levels of CS than poorer and lower educated women. In addition, both lower (<16 years) and higher (>35 years) maternal age have been shown to be associated with increased levels of CS [12,17-22]. Marital status and ethnic affiliation are other factors that influence the utilization of maternal health services [21,23,24]. Since men can be important decision-makers, paternal socio-demographic characteristics are also important in relation to health seeking behaviors and might influence birth outcomes [25-27].

According to the population-based Tanzania Demographic and Health Surveys (TDHS), about 50% of births in Tanzania occur in a health facility. Tanzania has had a low national CS level over the last decade, estimated to be 3% in 2004/05 and 5% in 2010. However, in the Kilimanjaro region in the Northern Zone of Tanzania, the CS level is more than double the national level, and it increased from 7.5% in 2005 to 11% in 2010 [27]. The Zonal tertiary hospital, Kilimanjaro Christian Medical Centre (KCMC) had a CS level of 33% in the years 2000 to 2007 [28].

Admittance and surgical procedures at KCMC involve user fees, and in the last decade the cost of delivery and CS have gradually increased [28]. More than 26% of all Tanzanians (2012) live below the poverty line [29] and difficulties in paying treatment is the most frequently reported barrier to health care in the Kilimanjaro region [27]. Although several groups in Tanzania are exempted from paying user fees, including those below a certain income level, this system is incompletely implemented, and private and faith-based hospitals often attempt to minimize the number of patients that are exempted [30]. Thus, although the CS percentage at KCMC is high, access may still be inequitable. Increasing user fees, combined with the closure of the operation theatre at the regional public hospital Mawenzi in 2010 may have resulted in poor women in the area having less access to CS than earlier. The aim of the study was to examine trends in the socio-demographic background of babies born at KCMC from the year 2000 to 2013, trends in the CS percentage, and to identify socio-demographic factors associated with CS at KCMC during this period.

## Methods

### Study design and data collection

This is a registry based study. The medical birth registry at KCMC was established in 1999 in collaboration with the University of Bergen, Norway. It has been in operation since July 2000 [31]. Information on birth outcome, delivery mode, obstetric history as well as socio- demographic factors is recorded in the registry [32]. Information is recorded by specially trained nurse-midwives using a questionnaire designed specifically for this purpose. The mothers are interviewed soon after recovery from the birth, usually within the first 24 hours, but later if complications occur. Supplementary information is collected from case files. Registration of this information is done every day, including weekends and holidays. A secretary then enters the data into an electronic file. Quality assurance of the birth registry has consisted of periodic instruction sessions [31]. The birth registry only registers deliveries at KCMC and includes both stillbirths and live births.

### Study area

The United Republic of Tanzania is the largest country in East Africa with about 45 million inhabitants (2012). Almost 75% of the inhabitants live in rural areas [33]. KCMC is one of four zonal/tertiary hospitals in Tanzania [34]. It is operated as a private/public partnership and located in Moshi town in the Kilimanjaro region. The region has more than 1.6 million inhabitants. Moshi rural district has a total population of 466,737 inhabitants whereas Moshi urban district has a population count of 184,292 [33]. KCMC has approximately 3300 deliveries per year, and the obstetric ward at KCMC receives high risk cases from seven regions in northern Tanzania and from some Kenyan districts [35]. In total the hospital thus serves more than 13 million people [36]. About 50% of the birthing women at KCMC come from Moshi urban district as they come for ordinary deliveries too [26]. About 20% of the birthing women come from Moshi rural district. The regional hospital, Mawenzi, also located in Moshi town, is supposed to offer emergency obstetric care for free, CS included, but the operation capacity has been relatively poor for a long time, and since December 2010, no CS have been conducted because the operation theatre closed. Thus KCMC has been the only referral institution that has offered CS after 2010 in Moshi, apart from private hospitals with substantial higher costs. About 88% of the women give birth in a health facility in Kilimanjaro [27].

The direct cost of normal delivery and CS at KCMC has gradually increased. Before 2005 the minimum price for CS was 20,000 TZS (=25.3 USD based on 01.01.2000 rates) but in 2005 a “cost sharing” policy was introduced and the out-of-pocket payment for CS was raised to a minimum of 50,000 TZS (=47.2 USD based on 01.01.2005 rates). It further increased to 100,000 TZS in 2011 (=58.4 USD based on 01.12.2011 rates) [37]. In addition to this the patients pay a per night fee, and pay for drugs and other costs associated with the hospital stay.

### Study population

There were a total of 45,871 births at KCMC in the period July 2000 to June 2013 of which 31,287 of the deliveries were among women residing in Moshi urban and Moshi rural districts. The majority of the deliveries, 29,752 (95.1%), were singleton births. We restricted the analyses to singletons born by women from the two Moshi districts (urban and rural) at KCMC hospital in the period July 2000 to June 2013.

### Description of variables

The main outcome variable was CS. The independent variables included education of the mother and the father, age of the mother, tribe of the mother, marital status of the mother, referral status, parity and year of delivery. Mother’s and father’s education completed were categorized into two categories: ‘no education/primary education’ (0–7 years) and ‘secondary/higher education’ (8 years or more). The variable maternal tribe was recoded as: ‘Chagga’, ‘Pare’ (which were the two most common), and ‘Other’, including more than 120 different tribes. Marital status was dichotomized as ‘married’ (i.e. monogamous and polygamous marriages or cohabitation) and ‘not married’ (i.e. separated/divorced, widowed or never-married). Age of the mother was included as a categorical variable with 4 categories: ‘13-17’ years, ‘18-25’ years, ‘26-35’ years and ‘36 to 47’ years. Women less than 13 years or more than 47 years of age were excluded in analyses including age as a variable. Parity was categorized in four: ‘para 1’, ‘para 2-3’, ‘para 4-6’ and ‘para 7+’. Referral status was divided into two categories: ‘medically referred’ (i.e. referred by qualified health personal for medical reasons) and ‘not referred’. Time of birth was included in most of the analyses as a continuous variable (called ‘year of birth’). However, in some of the analyses, time of birth was included as a categorical variable (called ‘time period’), with the categories representing three time periods associated with different levels of user fees at KCMC: Period 1: July 2000 to December 2004; period 2: January 2005 to November 2011; and period 3: December 2011 to June 2013.

### Statistical analysis

The data was analyzed using Statistical Package for Social Science (SPSS) version 20. All the analyses were stratified by mother’s residence (urban/rural). Frequency tables and graphs were used to describe changes in all births and CS deliveries year by year. Changes over time in the socio-demographic background of the babies born (all deliveries and CS) were tested using Chi square test for trend for each of the socio-demographic factors (with time both as a categorical variable with three time periods and as a integer variable: ‘year of birth’). Trends in the level of CS were examined using the Chi square test for trend, both overall and stratified by referral status. The likelihood of CS during the whole period was assessed using logistic regression. We started with bivariate analyses. We then developed models with interaction terms between each of the independent variables (socio-demographic factors and referral status) and time of birth as continuous variable. Finally, we developed a multivariable model with all the independent variables and significant interaction terms. Odds ratios with 95% confidence intervals were calculated. Babies with missing data on any of the independent variables (referral status, father’s and mother’s education level, marital status, mother’s tribe, mother’s age and parity) were excluded from the multivariable analyses.

### Ethical aspects

No person-identifiable information is available in the electronic birth registry handled by the researchers. Participation is based on verbal informed consent from the mothers. The birth registry at KCMC obtained ethical clearance from the Tanzania Ministry of Health, Commission for Science and Technology, from the KCM College and from the Norwegian National Ethics committee in 1999 [32]. The protocol for this study obtained ethical approval from Kilimanjaro Christian Medical University College of Tumaini University Makumira, in December 2013.

## Results

### Study population

The majority of the babies included in the analyses, 20,995 (71%), had mothers from urban areas. Most mothers were married and not medically referred. A higher proportion of fathers than mothers had secondary or higher education (Table 1). From time period 1 to time period 3, the proportion medically referred decreased from 14% to 12% amongst urban mothers and increased from 26% to 34% among rural mothers. The level of education of mothers and fathers increased, the proportion of single mothers increased, the age of mothers increased, the proportion Chagga mothers decreased and the proportion of mothers with high parity decreased significantly for all singleton babies born. Among babies born by CS, fathers’ and mothers’ educational levels increased, age of mothers increased, the proportion of married mothers decreased and the proportion medically referred decreased (Table 2). The same significant trends were found when using time of birth as a continuous variable (results not shown).

**Table 1:** **Characteristics of the study population and CS percentage, Moshi urban and rural districts**

**Singleton births**	**Moshi urban**			**Moshi rural**		
	**All births 2000–2013 n (%)**	**CS deliveries 2000–2013 n (%)**	**CS%**	**All births 2000–2013 n (%)**	**CS deliveries 2000–2013 n (%)**	**CS%**
**Referral status**						
Medically referred	2269 (11%)	1062 (19%)	47%	2423 (28%)	1187 (41%)	49%
Not referred	17938 (85%)	4318 (77%)	24%	5882 (67%)	1675 (54%)	29%
Missing	788 (4%)	211 (3.8%)		452 (5%)	137 (4.6%)	
**Father’s education level**						
No/primary education	8720 (42%)	2516 (45%)	29%	5192 (59)	1995 (67%)	39%
Sec/higher education	12223 (58%)	3063 (55%)	25%	3524 (40)	984 (33%)	28%
Missing	52 (0.2%)	12 (0.2%)		41 (0.5%)	20 (0.7%)	
**Mother’s education level**						
No/primary education	11362 (54%)	3125 (56%)	28%	6104 (70%)	2273 (76%)	37%
Sec/higher education	9602 (46%)	2460 (44%)	26%	2639 (30%)	722 (24%)	27%
Missing	31 (0.1%)	6 (0.1%)		14 (0.2%)	4 (0.1%)	
**Mother’s marital status**						
Married	18382 (88%)	4967 (89%)	27%	7623 (87%)	2607 (88%)	34%
Single	2562 (12%)	615 (11%)	24%	1084 (12%)	369 (12%)	34%
Missing	51 (0.2%)	9 (0.2%)		50 (0.6%)	23 (0.8%)	
**Mother’s tribe**						
Chagga	11613 (55%)	2905 (52%)	25%	5695 (65%)	1729 (58%)	30%
Pare	2299 (11%)	690 (12%)	30%	918 (11%)	419 (14%)	46%
Other	7058 (34%)	1991 (36%)	28%	2131 (24%)	845 (28%)	40%
Missing	25 (0.1%)	5 (0.1%)		13 (0.1%	6 (0.2%)	
**Mother’s age**						
13-17y	398 (2%)	97 (2%)	24%	280 (3%)	78 (3%)	28%
18-25y	7998 (38%)	1851 (33%)	23%	3416 (39%	1126 (38%)	33%
26-35y	10635 (51%)	3008 (54%)	28%	3954 (45%)	1405 (47%)	36%
36-47y	1931 (9%)	628 (9%)	33%	1092 (13%)	383 (13%)	35%
<13y, >47y	29 (0.1%)	7 (0.1%)		12 (0.1%)	5 (0.2%)	
Missing	4	0		3	2 (0.1%)	
**Mother’s parity**						
1	8843 (42%)	2152 (39%)	24%	3309 (38%)	998 (33%)	30%
2-3	9692 (46%)	2756 (49%)	29%	3725 (42%)	1410 (47%)	38%
4-6	2351 (11%)	663 (12%)	28%	1556 (18%)	544 (18%)	35%
7+	109 (0.5%)	20 (0.4%)	18%	167 (2%)	47 (1.6%)	28%
Missing	0	0		0	0

**Table 2:** **Characteristics of all births and CS deliveries in each period and linear trends, Moshi urban and rural districts**

**Singleton births**	**Urban Moshi**				**Rural Moshi**			
	**% of all births (% of CS deliveries)**			P-value* all births (CS deliv.)	**% of all births (% of CS deliveries)**			P-value* all births (CS deliv.)
	**Period 1**	**Period 2**	**Period 3**		**Period 1**	**Period 2**	**Period 3**	
**Referral status**								
Medically referred	14% (32%)	11% (17%)	9% (12%)	<0.001 (<0.001)	26% (44%)	30% (41%)	34% (39%)	<0.001 (0.096)
Not referred	86% (68%)	89% (83%)	91% (88%)		74% (56%)	70% (59%)	66% (61%)	
**Father’s education level**								
No/primary education	51% (55%)	40% (44%)	32% (33%)	<0.001 (<0.001)	64% (70%)	60% (69%)	50%(55%)	<0.001 (<0.001)
Sec/higher education	49% (45%)	60% (56%)	68% (67%)		36% (30%)	40% (31%)	50% (45%)	
**Mother’s education level**								
No/primary education	63% (65%)	54% (55%)	42% (43%)	<0.001 (<0.001)	76% (80%)	69% (77%)	57% (61%)	<0.001 (<0.001)
Sec/higher education	37% (35%)	46% (45%)	58% (57%)		24% (20%)	31% (23%)	43% (39%)	
**Mother’s marital status**								
Married	89% (89%)	88% (89%)	86% (88%)	<0.001 (<0.481)	89% (89%)	87% (88%)	84% (84%)	<0.001 (0.002)
Single	11% (11%)	12% (11%)	14% (12%)		11% (11%)	13% (12%)	16% (16%)	
**Mother’s tribe**								
Chagga	57% (51%)	55% (52%)	53% (53%)	<0.001 (0.228)	68% (60%)	64% (58%)	61% (54%)	<0.001 (0.015)
Pare	11% (12%)	11% (12%)	12% (13%)		11% (14%)	10% (14%)	11% (13%)	
Other	32% (37%)	34% (36%)	35% (34%)		21% (26%)	26% (28%)	28% (33%)	
**Mother’s age**								
13-17y	3% (3%)	2% (2%)	2% (1%)	<0.001 (<0.001)	3% (3%)	3% (3%)	2% (2%)	<0.001 (0.002)
18-25y	42% (40%)	37% (31%)	35% (29%)		42% (43%)	37% (34%)	39% (37%)	
26-35y	47% (49%)	52% (55%)	52% (56%)		43% (43%)	46% (49%)	47% (47%)	
36-47y	8% (8%)	9%(12%)	11% (14%)		11% (11%)	14% (14%)	12% (14%)	
**Mother’s parity**								
1	39% (39%)	43% (39%)	42% (37%)	<0.001 (0.653)	37% (34%)	37% (31%)	42% (40%)	<0.001 (0.116)
2-3	46% (48%)	46% (49%)	49% (51%)		42% (47%)	43% (49%)	42% (43%)	
4-6	14% (13%)	11% (12%)	9% (11%)		19% (17%)	18% (20%)	15% (17%)	
7+	1.1% (0.5%)	0.3% (0.4%)	0.3% (0.1%)		2.5% (2.4%)	1.7% (1.3%)	1.2% (0.6%)	

*Chi Square test.

**Figure 1:**
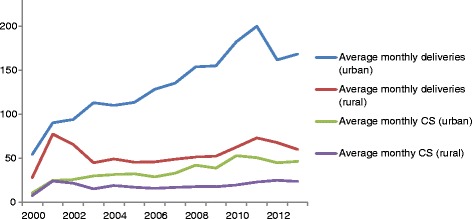
**Monthly average number of recorded singleton births and CS deliveries from July 2000 to June 2013 (Moshi urban and rural districts).**

The lowest mean monthly number of singleton births from Moshi was recorded in year 2000 with 83 deliveries. The monthly number of deliveries from the urban area increased from 55 in year 2000 to it peaked with 200 in year 2011. The monthly number of deliveries from the rural area remained stable (ranging from 45 to 53) from 2003 till year 2009. It then increased in year 2010 and peaked in 2011 with 73 deliveries. The mean monthly number of rural babies born by CS was stable in the period 2001 to 2013 (mean of 21). The mean monthly number of urban babies born by CS increased gradually from year 2000 to year 2010, ranging from 24 to 53 (Figure 1).

### Levels of caesarean section

**Figure 2:**
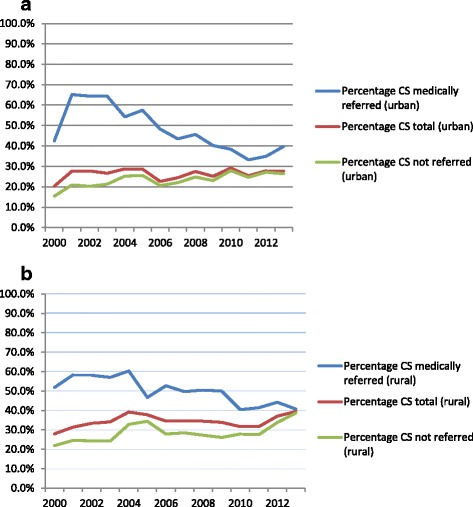
**Percentage of births that ended with CS among all mothers, medically referred and not referred mothers, 2000–2013. a**: Urban Moshi. Medically referred: p-value linear trend < 0.001, total: p-value linear trend = 0.640, not referred: p-value linear trend < 0.001. **b**: Rural Moshi. Medically referred: p-value linear trend < 0.001, total: p-value linear trend = 0.527, not referred: p-value linear trend < 0.001.

During the study period there were 8590 singleton deliveries by CS, giving an overall average percentage of 28.9%. The annual level of CS ranged between 20.3% and 29.1% for urban mothers and 27.9% and 39.4% for rural mothers (Figure 1). Stratification by referral status showed that the percentage of CS for both urban- and rural mothers decreased in the medically referred group (P-value for trend < 0.001) and increased in the group not referred (P-value for trend < 0.001) (Figure 2a and b). There was no linear trend in the overall CS percentage between 2000 and 2013. However, in the bivariate analyses, among urban babies there were significant two-way interactions between year of birth and the following variables: father’s education level (the likelihood of CS increased over time if father had no/primary education compared to if father had secondary/higher education), mother’s tribe (the likelihood of CS increased over time for Chagga mothers compared to mothers from other tribes), mother’s age (increasing likelihood of CS among those aged >25 years compared to those aged 18–25), parity (increasing likelihood of CS among para 4–6 compared to para 1) and referral status (decreasing likelihood of CS among medically referred compared to non-referred mothers). Amongst rural babies, there were significant two-way interactions between year of birth and referral status (decreasing likelihood of CS among medically referred compared to non-referred mothers). In the multivariable analysis, there were interactions between year of delivery and referral status for both urban and rural mothers. The odds of medically referred mothers having CS decreased with 12% (95% CI 10-15%) per year for those from urban Moshi and with 8% (95%CI 6-11%) for those from rural Moshi compared to non-referred mothers. Urban babies with fathers who had no or primary education had a relative 2% (95% CI 0.1%-4%) decrease in the odds of being born by CS per year compared to babies with fathers who had secondary or higher education. Urban Chagga mothers had a relative 3% (95% CI 1-5%) increase in the odds of CS per year compared to other tribes. Urban para 4–6 had a relative increase in the likelihood of CS per year of 6% (95% CI 2-10%) compared to para 1. Rural mothers had a 3% (95% CI 1-4%) increase in odds of CS per year (Table 3).

**Table 3:** **Bivariate and multivariable regression analyses of CS by background characteristics in pooled data from 2000–2013**

**Variable name**	**Caesarean section (urban)**				**Caesarean section (rural)**			
	**Crude OR**	**95%** **CI**	**Adjusted OR**	**95%** **CI**	**Crude OR**	**95%** **CI**	**Adjusted OR**	**95%** **CI**
**Year**	1.00	0.99-1.01	1.00	0.98-1.02	1.01	1.00-1.02	1.03	1.01-1.04
**Referral status**								
Medically referred	2.79	2.55-3.05	7.62	6.15-9.43	2.43	2.20-2.68	4.39	3.53-5.46
Not referred	Ref.	Ref.	Ref.	Ref.	Ref.	Ref.	Ref.	Ref.
Medically referred x year	0.87	0.85-0.89	0.88	0.85-.090	0.92	0.89-0.94	0.92	0.89-0.94
Not referred x year	Ref.	Ref.	Ref.	Ref.	Ref.	Ref.	Ref.	Ref.
**Father’s education level**								
No/primary	1.22	1.14-1.29	1.36	1.15-1.60	1.61	1.47-1.77	1.37	1.23-1.53
Secondary/higher	Ref.	Ref.	Ref.	Ref.	Ref.	Ref.	Ref.	Ref.
No/primary x time	0.98	0.96-0.99	0.98	0.96-0.997				
Secondary/higher x time	Ref.	Ref.	Ref.	Ref.				
**Mother’s education level**								
No/primary	1.10	1.04-1.17	0.96	0.89-1.04	1.58	1.43-1.75	1.17	1.04-1.32
Secondary/higher	Ref.	Ref.	Ref.	Ref.	Ref.	Ref.	Ref.	Ref.
**Mother’s marital status**								
Married	1.17	1.06-1.29	1.11	0.99-1.23	1.01	0.88-1.15	0.87	0.75-1.01
Single	Ref.	Ref.	Ref.	Ref.	Ref.	Ref.	Ref.	Ref.
**Mother’s tribe**								
Chagga	0.85	0.79-0.91	0.71	0.60-0.85	0.66	0.60-0.73	0.76	0.68-0.85
Pare	1.09	0.99-1.21	0.96	0.74-1.26	1.28	1.09-1.49	1.26	1.07-1.49
Other	Ref.	Ref.	Ref.	Ref.	Ref.	Ref.	Ref.	Ref.
Chagga x time	1.04	1.02-1.06	1.03	1.01-1.05				
Pare x time	1.01	0.98-1.04	1.01	0.98-1.04				
Other x time	Ref.	Ref.	Ref.	Ref.	Ref.	Ref.	Ref.	Ref.
**Mother's age**								
13-17y	1.07	0.85-1.35	0.90	0.54-1.50	0.79	0.60-1.03	0.61	0.45-0.82
18-25y	Ref.	Ref.	Ref.	Ref.	Ref.	Ref.	Ref.	Ref.
26-35y	1.31	1.22-1.40	1.23	1.02-1.49	1.12	1.02-1.23	1.26	1.12-1.42
36-47y	1.60	1.43.1.78	1.93	1.37-2.73	1.10	0.95-1.27	1.40	1.16-1.69
**Parity**								
1	Ref.	Ref.	Ref.	Ref.	Ref.	Ref.	Ref.	Ref.
2-3	1.24	1.16-1.32	1.03	0.85-1.24	1.41	1.28-1.56	1.35	1.20-1.53
4-6	1.22	1.10-1.35	0.57	0.42-0.78	1.25	1.10-1.42	0.98	0.83-1.17
7+	0.70	0.43-1.14	0.35	0.13-0.99	0.90	0.64-1.28	0.60	0.40-0.91
1 x time	Ref.	Ref.	Ref.	Ref.				
2-3 x time	1.01	0.99-1.03	1.01	0.98-1.03				
4-6 x time	1.05	1.02-1.08	1.06	1.02-1.10				
7 + x time	1.03	0.91-1.17	1.03	0.89-1.19				

### Socio-demographic factors associated with caesarean section

Having been medically referred, low levels of father’s and mother’s education, para 2–6 and higher maternal age were associated with higher likelihood of CS for both urban and rural residents in the bivariate logistic regression analyses. Amongst urban mothers, being married was associated with higher odds of CS compared to being single. In the multivariable model, having been medically referred for delivery was the factor which was most strongly associated with CS for both urban and rural babies, with ORs of 7.62 (95% CI 6.15-9.43) and 4.39 (95% CI 3.53-5.46) respectively. Low levels of father’s education and mother’s age 26–47 were also associated with higher likelihood of CS. Rural mothers with no- or primary education had higher odds of CS (OR 1.17, CI 1.04-1.32) and mothers aged 13–17 had decreased odds of CS (OR 0.61, CI 0.45-0.82) compared to mothers with secondary or higher education and age 18–25, respectively. Being Chagga and high parity (7+) were associated with lower likelihood of CS for both urban and rural mothers. Among rural mothers, being from the tribe Pare and para 2–3 were associated with higher odds of CS compared to other tribes and para 1, respectively (Table 3).

## Discussion

In this registry based study of 29,752 singleton deliveries from July 2000 to June 2013 at a large referral hospital in northeastern Tanzania, the average percentage of CS deliveries was 28.9%. There was no linear trend in the overall level of CS. During the 13 year period, the proportion of urban mothers who were medically referred for delivery decreased. The educational level and the age of the mothers went up for both urban and rural residents. As expected, the CS percentage was higher in the medically referred group than in the group not referred. For both urban and rural mothers low levels of education and maternal age 26–47 years were associated with higher likelihood of CS, whereas Chagga mothers and para 7+ had lower likelihood of CS compared to mothers from other tribes and para 1. Among rural residents, Pare mothers and para 2–3 had higher likelihood of CS compared to other tribes and para 1. There was no significant association between time and CS in the multivariable analysis for urban mothers but rural mothers had an increase in the odds of CS of 3% per year (95% CI 1-4%). There were significant two-way interactions between time and father’s education level, mother’s tribe and parity for urban mothers.

A CS percentage ranging from 20.3% to 29.1% for babies born by urban mothers, and 27.9% to 39.4% for babies born by rural mothers within a 13 year period is much higher than the Kilimanjaro region’s estimated population estimate of 11% (2010). The majority of women give birth in a health facility in Kilimanjaro [27]. Health facilities range from dispensaries to tertiary hospitals such as KCMC [30] and the high percentage of CS at KCMC might reflect the fact that few CSs are being performed at other health facilities in the region, implying that many women are referred to KCMC from lower level facilities due to medical complications. Several studies from Tanzania show that the quality of care in lower level facilities is insufficient [38-42] and that symptoms are often not acted upon properly at the stage of labour [43,44]. This might lead to preventable CSs [43] because it might be the only option left when the women arrive late at the referral facility. This would be in line with Worjoloh et al’s finding that labour dystocia and fetal distress are the second most common indications of CS at KCMC [34]. It is also likely that the high percentage of CS at KCMC includes unnecessary interventions as the leading cause of CS is reported to be previous CS [35]. Studies from SSA show that vaginal birth after one CS is safe if the health personnel have proper monitoring resources, knowledge and access to emergency CS [45-47]. Forceps and vacuum deliveries are less invasive and less expensive than CS, and carry lower risk in future pregnancies [14]. However, a low prevalence of operative vaginal delivery at KCMC [28] indicates that CS is the preferred solution when there is a high risk of complications during labour.

Different contexts make it difficult to compare the proportion of CS at institutions directly [1]. Nevertheless, before year 2000, facility CS levels in countries in SSA were reported to range from 5.0% to 21.8% [9]. Referral hospitals in East-African countries have more recently reported higher and increasing facility CS percentages than those seen at KCMC [48,49]. Little change was seen in the percentage of CS at KCMC in the study period even though there have been contextual changes. This could potentially be because there has been an increase in the proportion of deliveries among certain high risk women, but a decrease in another high risk group, like medically referred. With the closure of the operating theatre at the regional hospital Mawenzi, one could have expected increased levels, especially among the medically referred group. This was not seen, probably reflecting that few CSs were conducted at Mawenzi before the closure of the operating theatre. Similarly, the increases in user-fees in 2005 and in 2011 do not seem to have affected the overall percentage of CS. We did find that the likelihood of CS among referred women decreased with time, but this is difficult to explain without knowing whether referral practices have changed. The trends in CS level among all deliveries and among not referred deliveries are somewhat similar (Figure 2a and b). However, the increase for not referred women is greater, and this probably explains the discrepancy in p-values between all deliveries and not referred deliveries.

The finding that referral for delivery and low educational attainment were associated with CS, indicates that those in need and the relatively disadvantaged do have some access to CS when judged to be in need for it. It is likely that these sub groups are a selected group of women with higher risk of complications. It is logical that the level of CS among medically referred women is higher since they have been found to have a higher obstetric risk profile than those women that appear at the hospital without referral [28]. At the same time, high costs of services at KCMC may contribute to birthing women at KCMC being from more educated families than the average in the region: in period 1 (before 2005), 49% of all babies born and 45% of babies born by CS by urban mothers at KCMC were registered to have fathers with secondary or higher education, whereas in urban areas in the Kilimanjaro region the percentage of men age 15–49 with secondary or higher education was 15% in 2004/05 (unpublished data). The proportion of babies with lower educated mothers and fathers decreased significantly over time, possibly reflecting increasing economic barriers to seeking services at KCMC. The finding that babies with fathers who had no or primary education had a relative decrease in the likelihood of being born by CS per year compared to babies with fathers who had secondary or higher education may also reflect that fewer poor women seek health care at KCMC even when experiencing complications. However, changes in the socio demographic characteristics of the babies born at KCMC could also reflect transformations in the overall population. Repeated population-based surveys indicate that certain changes have occurred: the 2004/05 TDHS showed that 16.2% of the Kilimanjaro women had secondary or higher education [50], whereas this percentage increased to 27.7% in the 2010 TDHS [27]. Rural mothers are less represented at KCMC than urban mothers but have, like lower educated mothers or mothers with partners with little education, higher likelihood of CS. The higher levels of CS in these sub-groups might thus also reflect that women with little education or with partners with little education and rural women do not deliver at KCMC if they do not have or are expected to have complications. The finding that the likelihood of CS for rural mothers increased with 3% per year after adjustment for socio-demographic factors, despite the number of CSs from rural Moshi remaining stable, could reflect that birthing women from this district coming to KCMC were increasingly those with a high risk of more complicated delivery, while those with uncomplicated pregnancies delivered elsewhere.

Further investigation is needed to identify obstetrical risk factors in the different tribes to understand why Chagga women have lower likelihood of CS than other tribes. Unpublished data from KCMC indicate that Chagga women are normally of a higher stature and have lower BMI than Pare, and this might partly explain the lower CS percentage among Chagga. It is, however, not clear why the likelihood of CS increased with time for urban Chagga mothers and not for rural Chagga mothers. It is also possible that health seeking behavior could differ between the tribes, and this could affect the likelihood of CS.

Advanced maternal age is associated with different adverse pregnancy outcomes and higher risk of medical conditions like hypertension and diabetes [51] and this could explain why higher maternal age was associated with increased CS percentage at KCMC. It is difficult to explain why only mothers aged 13–17 from rural Moshi had lower probability of CS. The finding that the women giving birth at KCMC are getting older is probably reflecting a national trend (a higher age of first birth was reported in the TDHS in 2010 compared to 2004/05 [27,50]. Second and third time rural mothers (para 2–3) had a higher likelihood of CS compared to first-time mothers and this may reflect that rural women that delivered by CS in a previous pregnancy are asked to register at KCMC for the next birth to have a new CS. The lower likelihood of CS among urban mothers of para 4 or more in the multivariable model could reflect that many women with three CSs do not get pregnant again, in accordance with the standard medical advice given at KCMC: to avoid further pregnancies after three CSs. To understand why para 4–6 had an increased likelihood of CS over time, it would have been helpful to know the indications of CS. Very few women delivered for the seventh or more time, and the low OR of CS in this group may indicate that this is a highly selected group of low risk women.

### Strengths and limitations

A strength of this study is that the data was retrieved from a large birth registry where the data is collected systematically on a daily basis. Validation and quality checks of the database have been done, and the information has been deemed to be largely accurate [32]. The study sample was relatively large and covered a long time span. Because of the completeness of the data and the information on socio-demographic factors provided in the dataset, both regarding the mother and the father, we could assess how socio-demographic factors are related to CS in hospital settings over time. To our knowledge, very few studies in in SSA have done this before.

By using hospital based data, the study was limited to babies born at KCMC, and it does not give a complete picture of the differences in access to CS amongst the different socio-demographic groups in the Kilimanjaro region. There is a potential selection bias towards babies born by financially better off- and poor parents because of the cost sharing policy and the exemption system at KCMC. About 25% of all expected births from Moshi urban and only 4% of expected births from Moshi rural took place at KCMC in the period. The study is therefore not fully generalizable to all birthing women in Tanzania. We did not study medical indications for CS or reasons for referral which could have helped understanding the levels of CS better.

## Conclusions

The level of CS at KCMC remained high, but did not show the increasing tendency seen in similar hospitals in East Africa. Medical referral, lower educational attainment, and higher maternal age were associated with higher likelihood of CS. Women giving birth at KCMC, and their partners, are more educated than women and men of reproductive age in the Kilimanjaro region in general, and the educational level of the parents of babies born at KCMC increased over time, possibly reflecting inequitable access to the services offered at the hospital.

## Abbreviations

CS: Caesarean section
KCMC: Kilimanjaro Christian Medical Centre
MDG: Millennium development goal
SSA: Sub-Saharan Africa
TDHS: Tanzania Demographic and Health Survey
WHO: World Health Organization
